# Some History and Its Teachings

**Published:** 1917-08-18

**Authors:** 


### HOSPITAL SUNDAY IN LONDON.

### Some History and its Teachings.

Experience teaches that when the funds of a voluntary hospital or of an organisation like the Sunday Fund show signs of diminution year by year, or little or no advance, though the claims for help for the sick have a continuous upward tendency, it is certain there is something wrong with the *personnel* or the general management which is directly responsible for the administration of the institution. This experience is very instructive, and all who are interested in British charities should make a point of studying it.

The figures of the Metropolitan Hospital Sunday Fund will be found on pages 10 and 11 of the Report of the Council for 1916. They show that the receipts rose pretty steadily from £27,700 in 1873 to £45,331 in 1891. Then they dropped to under £40,000, but in 1895 a supreme effort to interest the public was made by The Hospital, with the result that the total increased to £60,361 in that year. It gradually went back, however, to £40,000, and then began to rise again in 1898, when the late George Herring gave his first donation of £10,000, and continued his gift of this sum—which he sometimes increased—until his death in 1906. In 1907 the Fund would have fallen to £45,000, or less, but for two legacies amounting to £33,000, of which £30,000 came from George Herring's estate. Since that date legacies, including that of George Herring, have varied from £37,000 to £22,221 each year, but the total receipts of the Fund fell to less than £65,000 in 1913, the contributions received from congregations being only £28,434, when the yield from this chief source of income was less than that in the second year of the Fund.

These figures manifested that something was wrong somewhere, and in a subsequent year circumstances brought about a change in the Vice-Chairmanship and the appointment of a new Secretary and Assistant Secretary. In 1915 the yield from contributions increased by over 33 per cent., and the total receipts to £75,646, of which £24,816 was from legacies. The vitality of the Hospital Sunday Fund has steadily progressed since. At the meeting of the Council on the 8th inst. the Vice-Chairman, Sir Ernest Tritton, in the absence of the Lord Mayor, had the gratification to announce that the amount to be distributed that day was the largest the Sunday Fund had ever had, with the exception of 1905, when a special legacy of £12,500 was included.

### Courage Brings Success.

We have often maintained that experience in the raising of money for our medical charities may be accepted as a leader which can be safely followed as a guide to the right conclusion of the causes, which, in the majority of cases, underlie a falling-off of funds. The Metropolitan Hospital Sunday Fund, as the figures we have given show, is a remarkable example of the force and truth of our contention that, if success is honestly desired, it requires an exercise of courage on the part of each governing body which must not fail when experience points to a change in the *personnel.* The Metropolitan hospitals and the citizens of London owe a deep debt of gratitude to Sir Ernest Tritton, the Vice-Chairman of the Sunday Fund, who took office in 1914, and has devoted himself to the interests of the Fund with a zeal and energy entailing the devotion of much time to the work. He is, in fact, deservedly a most popular Vice-Chairman, the proof of which is the steady and increasing yield of revenue, due to the carrying out of new schemes for raising funds and to the resulting and cordial co-operation of the clergy and ministers of all denominations, who have in increasing numbers shown great individual interest, with the result that the yield in 1917 has reached an amount which, in all the circumstances, is remarkable. Hearty thanks, as Sir Ernest Tritton said, are due to the clergymen and ministers who have put their heart into the work and directed many collections by bands of workers from people who are not in the habit of attending places of worship, as well as from the members of their congregations. The possibilities attached to the general acceptance of this new system may be gathered from the fact that in fifty places of worship, by the adoption of this scheme, the collections and offertories on Hospital Sunday, 1917, have been in many instances doubled, and sometimes even trebled. Sir Ernest Tritton declared that if all the clergy and ministers will combine to carry out the new plan, the income of the Fund may easily be quadrupled. If this be so, the income of Hospital Sunday in London each year may, and probably will, exceed £150,000 a year.

### London's Confidence in the Sunday Fund.

The magnificent record attained this year are a striking proof that the people of London still have confidence in the Hospital Sunday Fund, and that they appreciate the work the voluntary hospitals are doing for the sick poor of the Metropolis and for wounded sailors, soldiers, and airmen. The ever-increasing number of appeals in war-time for all sorts of objects, new and old, have exceeded those issued in any previous year in this country since the world began. All honour and grateful recognition, then, to those whose personal service has won back popularity and success for the Metropolitan Hospital Sunday Fund. In addition to the clergy and ministers and their co-workers throughout London, grateful recognition is especially due to Sir C. Ernest Tritton, Bart., and to Mr. Arnold James, and to Mr. J. A. R. Lander, the Secretary and Assistant Secretary of the Fund respectively. We anticipate this will be duly made at meetings of the Council and of the constituents respectively in December.

